# Combination of a novel oncolytic immunotherapeutic agent, CAVATAK (coxsackievirus A21) and immune-checkpoint blockade significantly reduces tumor growth and improves survival in an immune competent mouse melanoma model

**DOI:** 10.1186/2051-1426-2-S3-P125

**Published:** 2014-11-06

**Authors:** Darren Shafren, Min Quah, Yvonne Wong, Robert HI Andtbacka, Howard L Kaufman, Gough G Au

**Affiliations:** 1 Viralytics, Sydney, NSW, Australia; 2 School of BioMedical Sciences and Pharmacy, The University of Newcastle, New Lambton Heights, Australia; 3 Huntsman Cancer Institute, Salt Lake City, UT, USA; 4 Rutgers Cancer Institute of New Jersey, New Brunswick, NJ, USA

## 

Coxsackievirus A21 (CAVATAK™) is a bio-selected oncolytic immunotherapy virus. Following intratumoral (i.t) injection, CAVATAK selectively infects ICAM-1-expressing tumor cells, resulting in tumor cell lysis and a systemic immune-mediated anti-tumor response. A Phase II trial of i.t delivered CAVATAK (NCT01227551) in advanced melanoma patients has highlighted antitumor activity in both injected and distant non-injected lesions. Such responses have occurred at times when no circulating infectious CAVATAK was detected in patient serum and in an environment of high levels of anti-CAVATAK neutralizing antibodies. In further support of the generation of CAVATAK-mediated immune anti-tumor activity is the identification of a possible novel serum cytokine signature of elevated levels of IL-8 and IFN-γ in treated patients associated with tumor inflammation and systemic tumor response. Blockade of programmed death-1 (PD-1) in patients with metastatic melanoma has resulted in substantial tumor responses via a mechanism involving reversal of tumor-induced T cell suppression. We hypothesized that a combination of CAVATAK and PD-1 blockade may enhance anti-tumor responses, potentially leading to improved clinical activity. Preclinical studies in C57BL mice were conducted to assess the anti-tumor activity of CAVATAK and anti-mouse PD-1 (mPD-1) mAb in a B16-ICAM-1 melanoma immune competent mouse model. B16-ICAM-1 cells are murine melanoma B16 cells stably transfected to express human ICAM-1 allowing CAVATAK binding and cell infection. CAVATAK was administered i.t, while anti mPD-1 mAb was delivered intraperitoneally. Following treatment of the primary cutaneous B16-ICAM-1 tumor with 8 cycles of CAVATAK injections and 4 cycles of anti-PD-1mAb, mice were challenged with additional subcutaneous administration of B16 cells. Significant single agent anti-tumor activities against the primary B16-ICAM-1 tumor were observed in mice treated with either CAVATAK or anti-PD-1 mAb relative to saline controls. Combination of CAVATAK and anti-PD-1 mAb mediated significantly greater anti-tumor activity and offered greater survival benefit when compared to use of either agent alone. Of particular interest was the finding that a combination of CAVATAK and anti-PD-1 mAb was able to noticeably delay the onset of palpable tumor development following B16 cell challenge when compared to all other single agent treatment regimes. The significant anti-tumor activity mediated by the combination of CAVATAK and the checkpoint inhibitor antibody (anti-PD-1) observed in the presented murine melanoma model supports clinical evaluation of such an immunotherapeutic combination treatment regimen in patients with advanced melanoma.

